# Perfluoroalkyl Acid Binding with Peroxisome Proliferator-Activated Receptors α, γ, and δ, and Fatty Acid Binding Proteins by Equilibrium Dialysis with a Comparison of Methods

**DOI:** 10.3390/toxics9030045

**Published:** 2021-02-26

**Authors:** Manoochehr Khazaee, Emerson Christie, Weixiao Cheng, Mandy Michalsen, Jennifer Field, Carla Ng

**Affiliations:** 1 Department of Civil & Environmental Engineering, University of Pittsburgh, Pittsburgh, PA 15261, USA; MAK382@pitt.edu (M.K.); cheng_wx@pitt.edu (W.C.); 2 Department of Molecular and Environmental Toxicology, Oregon State University, Corvallis, OR 97330, USA; chriseme@oregonstate.edu (E.C.); jennifer.field@oregonstate.edu (J.F.); 3 U.S. Army Engineer Research Development Center—Environmental Lab, Vicksburg, MS 39180, USA; Mandy.M.Michalsen@usace.army.mil; 4 Secondary Appointment, Department of Environmental and Occupational Health, Graduate School of Public Health, University of Pittsburgh, Pittsburgh, PA 15261, USA

**Keywords:** equilibrium dialysis, peroxisome proliferator-activated receptors, fatty acid-binding proteins, per- and polyfluorinated alkyl substances, equilibrium dissociation constants

## Abstract

The biological impacts of per- and polyfluorinated alkyl substances (PFAS) are linked to their protein interactions. Existing research has largely focused on serum albumin and liver fatty acid binding protein, and binding affinities determined with a variety of methods show high variability. Moreover, few data exist for short-chain PFAS, though their prevalence in the environment is increasing. We used molecular dynamics (MD) to screen PFAS binding to liver and intestinal fatty acid binding proteins (L- and I-FABPs) and peroxisome proliferator activated nuclear receptors (PPAR-α, -δ and -γ) with six perfluoroalkyl carboxylates (PFCAs) and three perfluoroalkyl sulfonates (PFSAs). Equilibrium dissociation constants, K_D_s, were experimentally determined via equilibrium dialysis (EqD) with liquid chromatography tandem mass spectrometry for protein-PFAS pairs. A comparison was made between K_D_s derived from EqD, both here and in literature, and other in vitro approaches (e.g., fluorescence) from literature. EqD indicated strong binding between PPAR-δ and perfluorobutanoate (0.044 ± 0.013 µM) and perfluorohexane sulfonate (0.035 ± 0.0020 µM), and between PPAR-α and perfluorohexanoate (0.097 ± 0.070 µM). Unlike binding affinities for L-FABP, which increase with chain length, K_D_s for PPARs showed little chain length dependence by either MD simulation or EqD. Compared with other in vitro approaches, EqD-based K_D_s consistently indicated higher affinity across different proteins. This is the first study to report PPARs binding with short-chain PFAS with K_D_s in the sub-micromolar range.

## 1. Introduction

Per- and polyfluoroalkyl substances (PFAS) are widely used in a variety of industrial and consumer applications such as stain and water repellents, processing fluids, building blocks for fluoropolymers, and aqueous film-forming foams (AFFF) [1,2]. Various formulations of AFFFs containing short-chain PFAS continue to be used at military sites and airports to combat hydrocarbon-fueled fires, and their usage has resulted in persistent and widespread groundwater contamination [3,4,5]. AFFFs are complex mixtures containing high concentrations (g/L) of PFAS [6,7]. Polyfluorinated precursors in AFFF can degrade to form perfluorooctane sulfonate (PFOS), perfluorooctanoic acid (PFOA) and shorter-chain perfluoroalkyl carboxylates (PFCAs) [8,9], and perfluoroalkyl sulfonates (PFSAs) [10,11]. It is now recognized that many of the anionic forms (e.g., PFSAs and PFCAs) are highly persistent and mobile in the environment [12,13,14,15].

Biomonitoring has indicated these perfluorinated acids are generally found in highest concentrations in the blood plasma and liver [16,17,18], and are bound to proteins, as evidenced by both tissue distributions observed in laboratory and field studies and by targeted in vitro studies with isolated proteins or serum [19,20,21,22,23]. Relevant to these compartments are liver- and intestinal-fatty acid binding proteins (L-FABP and I-FABP), lipid-binding proteins highly expressed in the liver and intestine that play critical roles in binding, uptake, and transport of fatty acids [24]; and several subtypes (α, δ and γ) of peroxisome proliferator-activated receptors (PPARs), which serve as main transcriptional sensors of fatty acids and can control the expression of FABPs involved in fatty acid metabolism [25,26].

To date, only PPAR-α and -γ have been tested for binding with PFAS, and studies with FABPs have focused solely on the liver type [19,27,28]. Binding affinities for PFCAs (C4–C18) and PFSAs (C4–C8) were previously determined by fluorescence displacement methods with L-FABP [23,29,30] and PPAR-α [31]. There are no previously reported experimental data for PFAS binding to I-FABP or PPAR-δ, and only one for the ligand-binding domain (not the entire protein) of PPAR-γ [32]. Such studies show that long-chain PFAS, such as PFOS, and PFCAs with chain lengths between 9 and 12, bioaccumulate and bind with high affinity to serum proteins and liver fatty acid binding proteins (L-FABP). Less is known about PFAS binding to PPARs and how shorter-chain PFAS interact with biologically relevant proteins.

Because of the growing interest in the biological fate and effects of PFAS, experimental and modeling studies of PFAS-protein binding have proliferated. However, large differences persist across studies and across in vitro methods to assess binding, as well as between in vitro and modeling results. To date, the majority of PFAS-protein binding studies have focused on serum proteins, particularly human and bovine serum albumin [22]. In vitro studies with albumin [33] used a variety of methods including equilibrium dialysis [22,34,35,36,37], circular dichroism [38], NMR spectroscopy [22,32], ultrafiltration [39], surface tension [40], and electrophoresis [41]. Each technique has advantages and limitations, and lead to substantial differences in the binding affinities estimated. While ranking PFAS by chain length for relative protein binding affinity is well supported by both in vitro and in silico approaches for proteins such as serum albumin and L-FABP, there is little guidance on how to interpret the actual values obtained from the different approaches, which can differ by orders of magnitude [18,42,43]. It is, therefore, challenging to compare existing data for PFAS-protein binding or place modeling predictions into the context of experimental data.

Here, we employed a model-guided framework as an initial screen for the potential of both previously studied and of relevant but untested proteins (L-FABP, I-FABP, and PPARs α, δ, and γ) to bind with PFAS, followed by in vitro evaluation of predicted high-affinity PFAS–protein pairs. Model simulations, using molecular docking followed by molecular dynamics (hereafter referred to as MD), predicted the free energies of binding. The approach was based on our previous study, which demonstrated that MD can successfully predict relative protein binding affinity for L-FABP and PFCAs (C4–C9) and PFSAs (C4, C6, and C8) [42]. Here, our MD framework was used with new proteins to target potential high affinity binding to short-chain PFAS. Selected MD predictions were experimentally evaluated using equilibrium dialysis (EqD), which has been used previously to evaluate PFAS interactions with serum albumin [22,34], and is considered the gold standard for quantifying binding affinities [44]. Our EqD results were then compared with both MD predictions and with other available experimental data for protein binding with short-chain PFAS. We discuss similarities and differences among the different approaches for quantifying protein binding affinity, how results might be interpreted, and needs for further cross-validation.

## 2. Materials and Methods

### 2.1. Model-Based PFAS-Protein Affinity Screening

Initial selection of proteins for model-based screening was based on their known interactions with lipids and/or fatty acids, given the similarity between PFAS and these endogenous ligands [45,46,47]. The binding affinities between selected proteins and a total of five short-chain PFAS including perfluorobutanoic acid (PFBA), perfluoropentanoic acid (PFPeA), perfluorohexanoic acid (PFHxA), perfluoroheptanoic acid (PFHpA), and perfluorobutane sulfonate (PFBS) as well as four long-chain PFAS including PFOA, perfluorononanoic acid (PFNA), perfluorohexane sulfonate (PFHxS), and PFOS were estimated using the MD workflow developed by Cheng and Ng [42] with a goal to identify proteins that could have substantial binding affinity with short-chain PFAS. Briefly, three-dimensional (3D) structures were obtained from the Protein Data Bank (PDB, http://www.rcsb.org (accessed on 4 March 2020)) for L-FABP (PDB code: 3STM) [46], I-FABP (PDB code: 3AKM) [45], PPAR-α (PDB code: 4CI4) [48], PPAR-γ (PDB code: 3U9Q) [47], and PPAR-δ (PDB code: 3TKM) [49]. These proteins and nuclear receptors (Table 1) were selected because of their high structural resolution (<3Å) and their completeness, which is indicated by the inclusion of all amino acid residues that could be important to the protein binding sites in the structural model. The 3D structures for the PFCAs and PFSAs were either extracted from PDB (if available) or constructed from scratch using the Avogadro molecular editor [50], as previously described [42].

### 2.2. Experimental Assessment of Binding Affinity

#### 2.2.1. Materials

Linear PFBS, PFHxS, PFOS, PFBA, PFHpA, PFHxA, PFOA, and PFNA (all > 98% purity) were purchased from Wellington Laboratories (Guelph, Ontario, Canada). Purified human proteins L-FABP, I-FABP, PPAR-α, PPAR-γ, and PPAR-δ were obtained from Novus Biologicals (Littleton, CO, USA). Slide-A-Lyzer mini dialysis devices (10K MWCO, 0.1 mL) were purchased from Fisher Scientific (Hanover Park, IL, USA). Solvents (Fisher Scientific, Hanover Park, IL, USA) and other reagents were of analytical grade. All buffers were prepared from 10X phosphate-buffered saline from GIBCO Invitrogen (Grand Island, NY, USA). Dialysis materials were screened for PFCA and PFSA background and sorption prior to the onset of dialysis experiments. Material extraction analyses showed no concentrations of PFAS above the LOD (Table 2) within the dialysis cups or the dialysis tubes. Additionally, spiked water and equilibration experiments (24-h shake test) resulted in the recovery (75–235%) of PFAS analytes within the water, which indicated there was no level of detectable sorption of PFAS onto the dialysis cups or tubes. All other materials used in the processes were previously verified to have PFAS levels <LOD.

#### 2.2.2. Equilibrium Dialysis (EqD)

PFAS-protein binding affinities were evaluated by EqD. Experiments were conducted over a range of ligand: protein mole ratios (0.05, 0.1, 0.5, 1, and 5). These mole ratios represent concentrations ranging from 0.33 to 153.5 ng/mL, depending on the PFAS. In general, the average levels of PFSA and PFCA in plasma of people living in urban areas are about 20 ng/mL and 10 ng/mL, respectively (e.g., [51,52,53,54,55,56,57,58]). It should be mentioned some studies report higher concentrations of PFAS (between ~60 and 100 ng/mL) in the plasma of people living near fluorochemical plants, airports, and/or military sites [59,60]. The selected mole ratio ranges therefore encompass the expected concentrations found in human plasma. For all PFAS, 10 µM stock solutions were prepared by dissolving each chemical in 18.1 mS/cm phosphate-buffered saline, which was achieved by diluting the stock buffer tenfold with deionized water to give a solution that was pH 7.4. Stock solutions of different proteins were prepared fresh daily in phosphate buffered saline. Specific PFAS and protein concentrations were selected to achieve a 1:1 PFAS to protein molar ratio at the midpoint of the range of selected PFAS concentrations. Protein concentrations in prepared solutions were verified using the Qubit Protein assay kit (Thermo Fisher, Waltham, MA, USA).

EqD experiments were performed at room temperature by first adding 1.2 mL of the 18.1 mS/cm phosphate buffered saline (pH 7.4) spiked with PFAS to a 1.5 mL polypropylene microcentrifuge tube (Supplemental Figure S1). A Slide-a-Lyzer mini dialysis cup containing a semi-permeable membrane (molecular weight cutoff: 10kDa) was then inserted into the tube, through which PFAS could freely pass but which was impermeable to the proteins used (MW range 15.1–54.1 kDa). A known volume of protein in buffer (20 to 50 μL) was added to reach a 1 μM concentration for L-FABP, I-FABP, and PPAR-γ, and 0.48 μM for PPAR-δ and PPAR-α. The lower concentration of PPAR-δ and PPAR-α was necessary due to the larger size of these proteins. Finally, the total volume in the dialysis cup was brought to 100 μL by adding the buffer spiked with PFAS.

Blanks were prepared using a protein solution with no PFAS. Non-binding controls (containing PFAS but no protein) were prepared with the buffer spiked with different concentrations of PFAS. Finally, samples were placed on a rocker (Open-Air Rocker, Fisher Scientific, Waltham, MA, USA) for 36 h to reach equilibrium at room temperature. All dialysis tests were performed in duplicate.

### 2.3. Analysis by LC-MS/MS

All dialysate samples were analyzed without dilution or first diluted into water to reach concentrations of 100–2000 ng/L prior to analysis. Final sample volumes (1.5 mL) were spiked with 24 μL of isotopically labeled internal standards for quantification prior to injection. A modified Agilent 1100 series HPLC (Santa Clara, CA, USA) was used for large volume (900 μL) injection of aqueous samples. A C18 (4.6 × 50 mm × 5 μm Zorbax Eclipse, Agilent, Santa Clara, CA, USA) delay column was used between the LC pump and autosampler to separate out instrumental background. Retention of analytes was achieved with a C18 analytical column (Eclipse 4.6 × 100 mm × 3.5 μm, Agilent, Santa Clara, CA, USA) and mobile phases were 20 mM ammonium acetate in HPLC-grade water (A) and HPLC-grade methanol (B). A ten min LC gradient was used as follows: mobile phase A at 0.5 mL/min for 3.5 min, mobile phase B at 1 mL/min for 1.5 min, and mobile phase A at 1.0 mL/min for 4.5 min reduced to 0.5 mL/min for the remaining 0.5 min.

Identification and quantification of analytes were previously described in Allred et al. [61]. The analytical sequence consisted of a minimum 5-point calibration curve over the range of 20–10,000 ng/L for all analytes. Accuracy was determined from the analysis of a second source of standards and were required to be 70–130% of the target value. Whole method precision, as indicated by relative standard deviation, was calculated from four replicate samples, and ranged from 4 to 18%. The limit of detection (LOD, 6 ng/L) was calculated by normalized-weighted regression (1/X), from which the limit of quantification (LOQ) (20 ng/L) was calculated as 3.3 × the LOD [7]. Each analytical sequence consisted of solvent blanks that were spiked with 24 μL of isotopically labeled standards; all blanks gave responses that fell below the LOQ.

**Figure 1:**
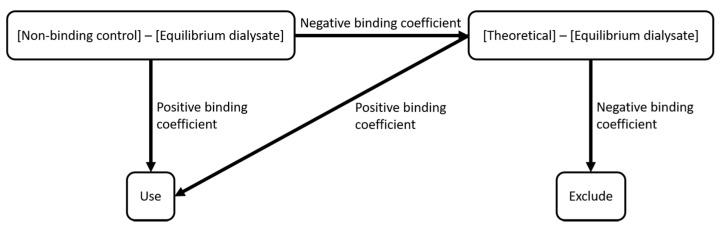
Decision tree for the inclusion of the equilibrium dialysate concentrations for the regression analysis.

Binding coefficients for protein-PFAS pairs were calculated from the difference in PFAS concentrations (mole ratio) between the non-binding control and equilibrium dialysates. Data for all dialysis experiments were analyzed by nonlinear regression, assuming a single-site binding model using GraphPad Prism V8.1.2 (GraphPad software, San Diego, CA, USA) to determine K_D_ [62,63,64,65]_._ Some EqD concentrations, when subtracted from the non-binding control, produced a negative binding coefficient indicating a final equilibrated concentration greater than the initial dialysate concentration. As both the EqD experiment and non-binding control come from the same stock, the EqD concentration should, at most, equal that of the non-binding control. This may have been an artifact of dilution; at high initial concentrations, 15 to 3000-fold dilutions were required to bring PFAS on-scale for detection. In cases where large dilution factors were required, uncertainty about the calculated final concentrations in the dialysate may be magnified. In order to better address this, a decision tree was created to determine the handling of these incidents (Figure 1).

### 2.4. Comparison to Existing PFAS-Protein K_D_s and Methods

In order to place our results in context with existing literature and provide insight into in vitro and modeling choices, we conducted a literature search for all available PFAS-protein binding data that used the same proteins as investigated here. In addition, we screened existing serum albumin studies that used equilibrium dialysis, where the results could be compared across different methods as done here for FABPs and PPARs. The search spanned publication years between 1954 and 2020, and resulted in 37 studies used for comparison of methods.

## 3. Results and Discussion

### 3.1. Screening Protein–PFAS Pairs by Molecular Dynamics

Molecular dynamics modeling predicted free energies of binding which, when converted to equilibrium dissociation constants (K_D_ values), ranged between approximately 10^−5^ and 10^6^ μM, corresponding to femtomolar to molar dissociation constants. Relevant interactions with and between biomolecules occur at a range of dissociation constants from low millimolar (the weakest) to femtomolar (the strongest) [66]. It is generally accepted that the most biologically relevant (moderate to strong) interactions correspond to K_D_ values at micromolar levels and lower [67]. This suggests that predicted binding affinities, if assumed to be similar to in vivo binding affinities, are unlikely to be biologically relevant if the K_D_ values are substantially larger than 10^3^ μM.

**Figure 2:**
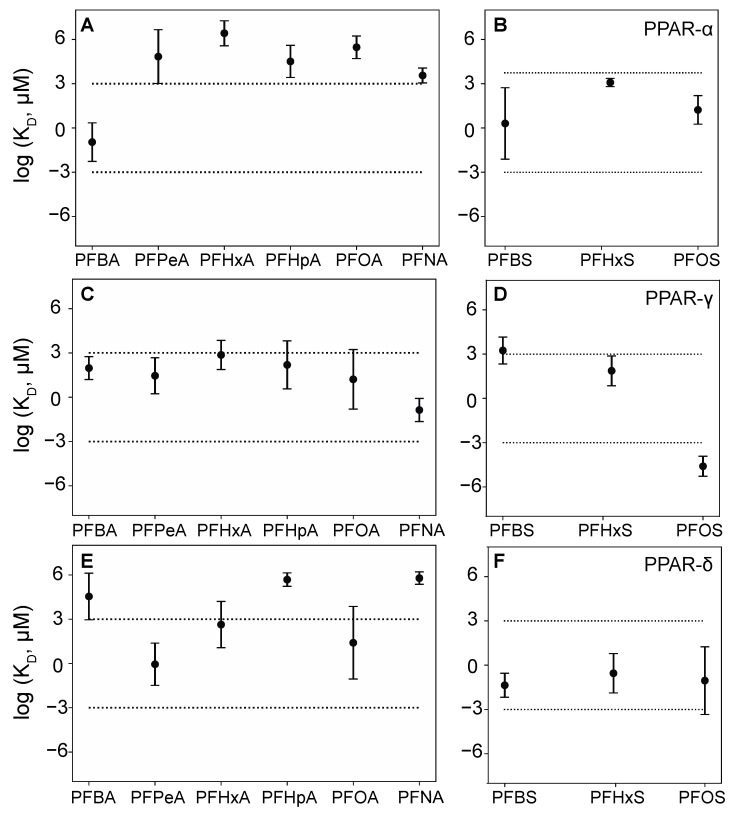
Predicted dissociation constant (K_D_) values (geometric mean ± 1 standard error) for different peroxisome proliferator-activated receptors (PPAR)–per- and polyfluorinated alkyl substances (PFAS) complexes. (**A**) PPAR-α and perfluoroalkyl carboxylates (PFCAs) (**B**) PPAR-α and perfluoroalkyl sulfonates (PFSAs) (**C**) PPAR-γ and PFCAs (**D**) PPAR-γ and PFSAs (**E**) PPAR-δ and PFCAs (**F**) PPAR-δ and PFSAs. Values of log K_D_ > 3 correspond to millimolar or weaker binding, between −3 and 3 are moderate (in the micromolar range) and < −3 correspond to strong, nanomolar, or lower binding.

Based on the MD predictions, we selected fifteen PFAS–protein pairs to experimentally determine K_D_ values using equilibrium dialysis (Supplemental Table S1). We selected the short-chain PFCA PFBA for EqD testing with PPAR-α because of its strong predicted affinity (Figure 2A); PFHxA, PFHpA, and the long-chain PFNA were selected for EqD testing with PPAR-α as well. This range allowed us to evaluate both the surprising prediction of strong affinity for PFBA and the predicted lack of chain length dependence for the PFCAs experimentally, particularly given the lack of other experimental data. For PPAR-γ, since no short-chain PFAS were predicted to bind strongly, we selected only PFOA and PFOS for EqD testing. For PPAR-δ/β, we selected the three sulfonates, PFBS, PFHxS, and PFOS. This allowed us to verify, first, the strong predicted binding with PFBS and, second, the counterintuitive chain length dependence predicted by MD for the sulfonates.

**Figure 3:**
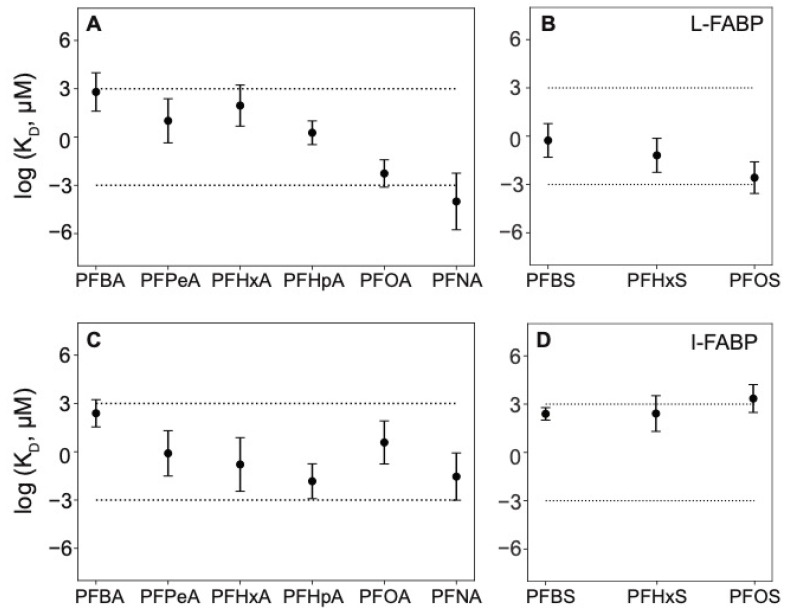
Predicted dissociation constant (K_D_) values (geometric mean ± 1 standard error) for (**A**) liver fatty acid binding proteins (L-FABP) and PFCAs, (**B**) L-FABP and PFSAs, (**C**) intestinal fatty acid binding proteins (I-FABP) and PFCAs, and (**D**) I-FABP and PFSAs. Values of log K_D_ > 3 correspond to millimolar or weaker binding, between −3 and 3 are moderate (in the micromolar range) and <−3 correspond to strong, nanomolar or lower binding.

The relatively well studied L-FABP provides an opportunity to compare with multiple other studies, both modeling and in vitro. For L-FABP, PFOS was selected for EqD testing because it was predicted to have the strongest binding affinity (Figure 3B); PFOA and PFHxS were selected as well to compare the effect of the head group (carboxylate vs. sulfonate). For evaluating potential binding with short-chain PFAS, only PFBS has moderately strong predicted binding affinity (compared to carboxylates). For I-FABP, PFHpA, and PFNA showed the strongest binding and were therefore selected. Further discussion regarding MD results can be found in the SI. Mean serum levels of PFBA, PFHxA, PFOA, PFNA, PFBS, and PFOS have been documented in humans living near industrial and urban areas at about 0.9 ng/mL, 0.1 ng/mL, 4 ng/mL, 0.8 ng/mL, 0.1 ng/mL, and 23 ng/mL, respectively (e.g., [68,69,70,71,72,73]).

### 3.2. EqD-Based Dissociation Constant (K_D_) Estimates

#### 3.2.1. PPAR-α

**Figure 4:**
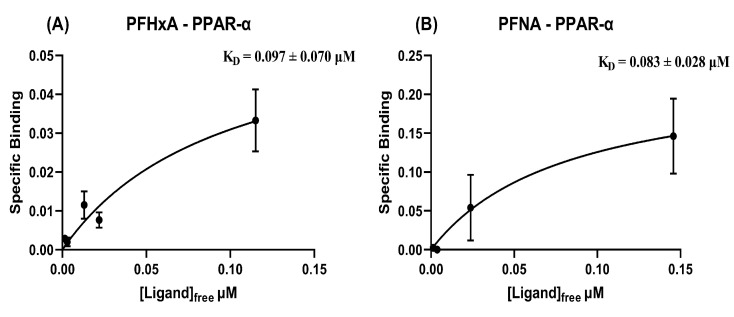
Specific binding (μmol PFAS/μmol protein) vs free concentration of PFAS (μmol/L), used for nonlinear fit of K_D_ (in μM, ± S.E.) for (**A**) PFHxA and (**B**) PFNA with PPAR-α.

Strong binding for PFHxA (Figure 4A) and PFNA (Figure 4B) were observed via EqD experiments, whereas no binding occurred for PFBA (Supplemental Figure S2A) and PFHpA (Supplemental Figure S2B). The lack of chain length dependence suggested by this is in agreement with the MD predictions. However, MD simulations suggested only PFBA would have strong binding for PPAR-α, which was not borne out by dialysis. The relatively strong binding suggested by the K_D_ of 0.097 μM for PFHxA could have implications for short-chain PFAS safety.

#### 3.2.2. PPAR-γ

Strong binding was found between PFOA and PPAR-γ (Supplemental Figure S3) which agrees with previous experimental evidence that PFOA is a PPAR-γ activator [74]. Additionally, PFOS binds to PPAR-γ, albeit with substantially lower affinity. These EqD-derived K_D_ values are the first reported for PPAR-γ with PFOA and PFOS. MD binding predictions were in agreement with observed K_D_ values for both PFOA and PFOS (Figure 2C,D).

#### 3.2.3. PPAR-δ

**Figure 5:**
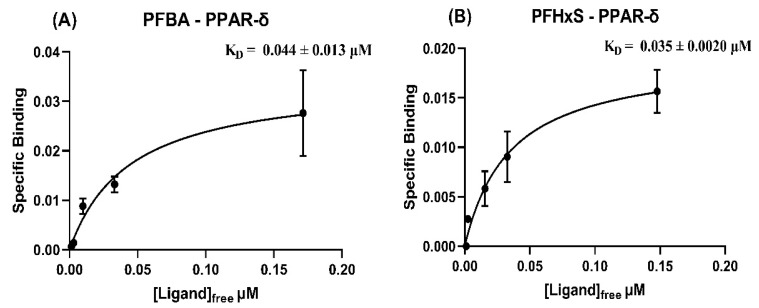
Specific binding (μmol PFAS/μmol protein) vs free concentration of PFAS (μmol/L), used for nonlinear fit of K_D_ (in μM, ± S.E.) for binding affinity of (**A**) PFBA and (**B**) PFHxS with PPAR-δ.

Strong binding to PFBA, PFHxS, and PFOS (Figure 5 and Supplemental Figure S4) was observed for the first time with this protein. Like PPAR-α, PPAR-δ also had measurable binding to a short chain PFCA (PFBA) and did not adhere to the increased binding affinity with increasing chain length trend observed for L-FABP. Again, this indicates that short-chain PFAS safety based on body clearance alone may not be reliable, and more research into the interactions that may occur during clearance is warranted. Additionally, chain length, while generally a good indicator of PFAS retention in a system, may not be an indicator of binding affinity to any given protein. Detectable binding affinities for PPAR-δ were in the range of 10^−2^ to 10^−1^ μM. MD simulations were in agreement for PFHxS and PFOS. However, predicted binding to PFBS was not detected experimentally, whereas experimental binding to PFBA was observed but not predicted (Figure 2E,F). Overall, PPAR MD simulations were effective in identifying relative binding affinities and provided confidence in selection of PFAS-protein combinations but are not currently able to predict absolute affinity.

#### 3.2.4. L-FABP

**Figure 6:**
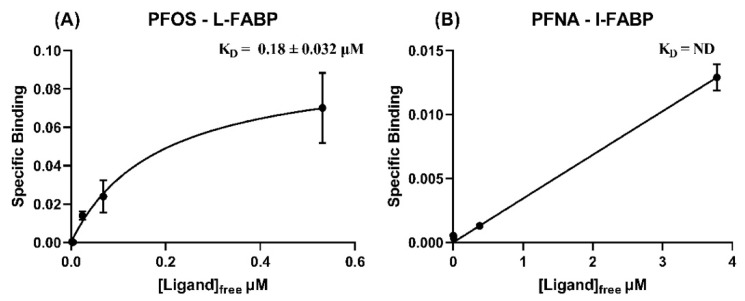
Specific binding (μmol PFAS/μmol protein) vs free concentration of PFAS (μmol/L), used for nonlinear fit of K_D_ (in μM, ± S.E.) for binding affinity of (**A**) PFOS with L-FABP and (**B**) PFNA with I-FABP.

Our EqD results for L-FABP generally agreed with previous observations in terms of relative affinities. That is, binding was strongest for the long-chain PFAS tested, PFOA and PFOS (0.099 and 0.18 μM, respectively, see Figure 6A for PFOS and Supplemental Figure S5A for PFOA), weaker for PFHxS (1.7 μM, Supplemental Figure S5D), and not detected for the shortest PFAS tested, PFHxA and PFBS. Experimentally derived KD values for PFOS, PFHxS, and PFOA fell within the range of model predictions (Figure 3A,B).

These are the first experimental data for PFCAs binding to I-FABP. Molecular dynamics results for I-FABP indicated PFHpA and PFNA should both demonstrate relatively strong binding (Figure 3C). However, no binding was detected by EqD for either PFHpA or PFNA (Figure 6B and Supplemental Figure S6) and therefore no K_D_ values could be determined (Table 3).

Since these are the first experimentally determined K_D_s for I-FABP, there are no other studies to aid in evaluating whether the MD simulations or dialysis results are more problematic. The MD results of PFSAs indicated very weak interactions for all chain lengths, which is more in line with the dialysis observations for the PFCAs tested.

### 3.3. Comparison Across In Vitro Methods to Evaluate Binding

**Figure 7:**
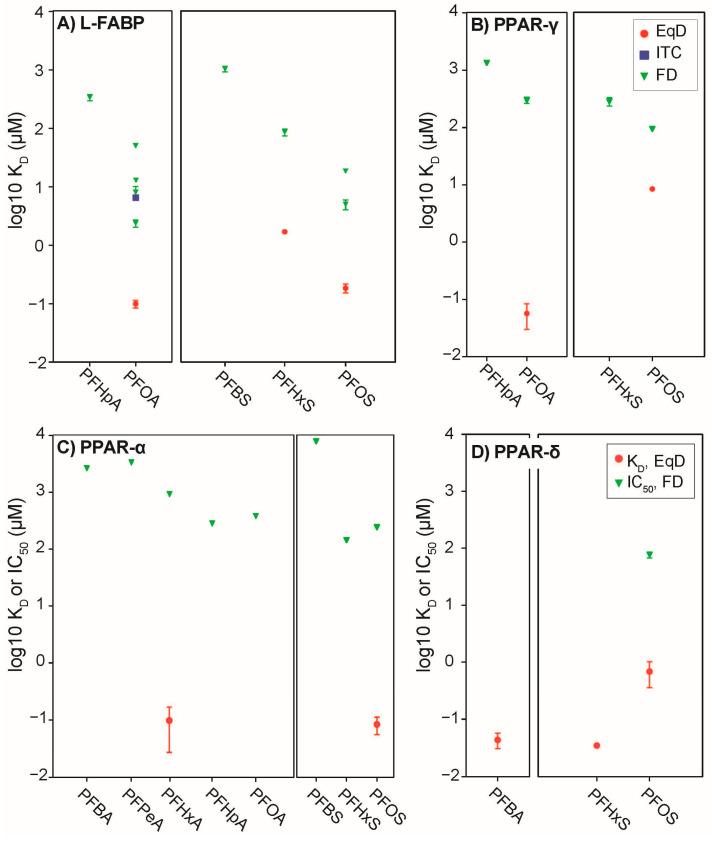
Comparison of K_D_s for PFAS with eight or fewer fluorinated carbons measured by equilibrium dialysis (EqD) in this study (red symbols) compared with (**A**) K_D_ measured by fluorescence displacement (FD) and isothermal titration calorimetry (ITC) for L-FABP, (**B**) K_D_ measured by FD for PPAR-γ, and (**C**) IC_50_ (right axis) measured by FD for PPAR-α and (**D**) PPAR-δ.

Comparison of experimentally derived K_D_ values from this and previous studies suggest that EqD consistently generates lower K_D_ values (stronger binding affinities) than other approaches. Fluorescence displacement has recently emerged as a widely applied method to measure protein binding affinity [75]. Fluorescence displacement is a convenient and relatively high-throughput approach but, as shown here, will consistently indicate lower affinity binding that EqD (Figure 7 and Supplemental Figure S1; Tables S2 and S3). For L-FABP, observed K_D_ values from this study were substantially lower than previously published values (Figure 7A) [23,76]. Experimentally derived K_D_ values for PFOA and PFOS with PPAR-γ were lower than those reported by Zhang et al. [32], three to four orders of magnitude in the case of PFOA and one order of magnitude for PFOS (Figure 7B). K_D_ values for PFHxA and PFNA with PPAR-α measured by equilibrium dialysis are lower than those reported by Ishibashi et al. [31] by several orders of magnitude (Figure 7C). Although Ishibashi et al. [31] report 50% inhibitory concentrations (IC_50_) rather than K_D_, the magnitude of the differences between results is unlikely to be attributable to this. The IC_50_ in the case of the Ishibashi et al. [31] study describes the concentration of the competitor (i.e., PFAS) at which 50% of the fluorescent molecule was displaced, and is thus an indirect measure of binding affinity. IC_50_ may vary according to the competition regime and experimental conditions, but for competitive inhibition (i.e., displacement by PFAS from the same binding site) should be of similar magnitude, as these values are linked by ligand and substrate concentrations. Similar to results for PPAR-α, Li et al. [77] reported competitive binding based IC_50_ for PPAR-δ with PFBA, PFHxS, and PFOS, wherein only PFOS showed detectable binding (Figure 7D). EqD-determined binding coefficients in this study for PFBA, PFHxS, and PFOS with PPAR-δ were lower than those reported IC_50_ values, with PFBA and PFHxS in particular showing strong binding.

Similar observations have been made before, for example between EqD and ^19^F-NMR and micro-size exclusion chromatography for serum albumins [22]. A literature search comparing methods to determine binding for human serum albumin (HSA) and bovine serum albumin (BSA) also showed EqD to consistently produce lower K_D_ values than other methods (Supplemental Figures S7 and S8 and Table S3). This indicates that the low K_D_ values measured here are not an artifact of this study but rather a consistent outcome of the EqD approach.

## 4. Conclusions

This is the first study to report short chain PFAS-PPAR binding with K_D_s in the sub-micromolar range, raising the possibility that short-chain replacements for long-chain PFAS may still be bioactive, despite the assumed “safety” of short-chain PFAS on the basis of rapid serum clearance [67]. PPARs are nuclear receptors that play critical roles in the regulation of many biological processes, including cell growth, lipid metabolism, differentiation, and inflammation [78]. Previous in vitro and in vivo studies have reported that both PFCAs and PFSAs can activate PPAR-α and PPAR-γ [15,32,79], but have not found activation of PPAR-δ [28]. This is the first study to report strong interactions with PPAR-δ and PFCAs having fewer than seven perfluorinated carbons. The lack of chain length dependence we observed with PPAR-α and PPAR-δ by both MD simulations and EqD indicates that PFAS binding affinity to proteins should not be inferred by PFAS carbon chain length for all proteins, but is rather specific to the protein being considered.

Despite the accumulating data, there is a persistent lack of clarity on how either modeling or in vitro studies relate to the behavior of PFAS in vivo, within natural biological and environmental contexts—that is, in competition with native ligands and other environmental contaminants. EqD may indicate higher binding affinity because it measures binding in a highly controlled system independent of other factors. In vivo, competitive interactions are more likely to be the dominant mode. That being said, it is still unclear whether typically used fluorophores are at all representative of native ligands and other xenobiotics that make up the real-world competitors of PFAS for protein sites. Thus, a competitor-agnostic approach, such as equilibrium dialysis, may still be preferable. Moreover, consistently lower K_D_ values across many different proteins raises an important question that is yet to be answered and will be key for making reliable in vitro to in vivo extrapolations: do the lower K_D_s indicate the EqD approach is capable of quantify binding that other approaches do not? If so, this could suggest that binding affinities of PFAS to proteins considered here, and possibly other proteins, have been historically underestimated, and subsequent research using data from different approaches should recognize that EqD generates lower K_D_ values.

In some cases, it is possible that MD simulations could be improved by longer simulation times. However, increasing the simulation time from 24 ns to 45 ns for all of the PPAR-PFAS combinations presented here would require months of additional computation time. Therefore, when undertaking and interpreting these modeling approaches it is important to acknowledge the time resource component. The comparison of modeled and experimentally determined values in this study further confirms our previous observation [42] that MD simulations are best for predicting relative rather than absolute K_D_ values. The extent of agreement between measured and modeled values varied substantially among proteins, but chain length dependencies or lack thereof were generally consistent. Additionally, MD simulations predict stronger binding than is experimentally observed through fluorescence displacement but weaker binding than may be observed via equilibrium dialysis. Future research is needed to understand how different binding values relate to in vivo consequences and if any particular method should be used for in vitro to in vivo extrapolation.

## Figures and Tables

**Table 1:** Summary of 3-dimensional structure information for selected proteins.

Protein *	PDB Code	Resolution	Chain Length	Known Ligands
L-FABP	3STM	2.22 Å	132	palmitic acid
I-FABP	3AKM	1.9 Å	131	11-(Dansylamino) undecanoic acid
PPAR-α	4CI4	2.3 Å	274	propanoic acid
PPAR-γ	3U9Q	1.5 Å	269	decanoic acid
PPAR-δ	3TKM	1.95 Å	275	GW0742

* Liver and intestinal fatty acid binding proteins (L-FABP, I-FABP); peroxisome proliferator-activated nuclear receptors, isoforms α, γ and δ (PPAR- α, γ, and δ).

**Table 2:** Dialysis material extraction and sorption results.

Material Extracts	PFBA	PFHxA	PFHpA	PFOA	PFNA	PFBS	PFHxS	PFOS	Surrogate Recovery
Collection tube	<LOD	<LOD	<LOD	<LOD	<LOD	<LOD	<LOD	<LOD	89%
Recover tube	<LOD	<LOD	<LOD	<LOD	<LOD	<LOD	<LOD	<LOD	90%
Dialysis membrane	<LOD	<LOD	<LOD	<LOD	<LOD	<LOD	<LOD	<LOD	89%
Dialysis cap	<LOD	<LOD	<LOD	<LOD	<LOD	<LOD	<LOD	<LOD	91%
Sorption to Materials									
2000 ng/L Spike 1	5700	2700	3200	3300	2600	2600	2030	2700	
2000 ng/L Spike 2	2500	1500	2100	2600	1900	1700	2600	2400	
% Recovery 1	285%	135%	160%	165%	130%	130%	101%	135%	
% Recovery 2	125%	75%	105%	130%	95%	85%	130%	120%	

**Table 3:** Dissociation constant (K_D_) values ± SE measured by equilibrium dialysis.

Protein	PFAS	K_D_ (µM)
L-FABP	PFHxA	ND
	PFOA	0.099 ± 0.015
	PFBS	ND
	PFHxS	1.7 ± 0.031
	PFOS	0.18 ± 0.032
I-FABP	PFHpA	ND
	PFNA	ND
PPAR-α	PFBA	ND
	PFHxA	0.097 ± 0.070
	PFHpA	ND
	PFNA	0.083 ± 0.028
PPAR-γ	PFOA	0.057 ± 0.027
	PFOS	8.5 ± 0.46
PPAR-δ	PFBA	0.044 ± 0.013
	PFBS	ND
	PFHxS	0.035 ± 0.0020
	PFOS	0.69 ± 0.33

“ND”: no dissociation constant could be determined, indicating low to no binding.

## Data Availability

The data presented in this study are available in the article and in the supplementary material here or were extracted from published studies (for method comparisons).

## Supplementary Materials

The following are available online at https://www.mdpi.com/2305-6304/9/3/45/s1, Figure S1. Equilibrium dialysis setup with materials used (dialysis filters and vials) shown. Figure S2. Equilibrium dialysis results for binding affinity of PFBA (A) and PFHpA (B) with PPAR α with pH = 7.4 and ionic strength = 18.1 mS/cm. Figure S3. Equilibrium dialysis results for binding affinity of PFOA (A) and PFOS (B) with PPAR-γ with pH = 7.4 and ionic strength = 18.1 mS/cm. Figure S4. Equilibrium dialysis results for binding affinity of PFOS (A) and PFBS (B) with PPAR-δ with pH = 7.4 and ionic strength = 18.1 mS/cm. Figure S5. Equilibrium dialysis results for binding affinity of PFOA (A), PFBS (B), PFHxA (C), and PFHxS (D) with L-FABP with pH = 7.4 and ionic strength = 18.1 mS/cm. Figure S6. Equilibrium dialysis results for binding affinity of PFHpA with I-FABP with pH = 7.4 and ionic strength = 18.1 mS/cm. Figure S7. Comparison of reported KD (± SE) values from literature for human serum albumin [15,16,17,18,19,20,21,22,23,24]. Figure S8. Comparison of reported KD (± SE) values from literature for bovine serum albumin [15,20,24,25,26,27]. Table S1. Matrix of selected protein-PFAS combinations for batch analysis. Table S2. Comparison of methods L- and I-FABP and PPAR α, γ, δ [8,9,28,29,30,31,32]. Table S3. Comparison of methods HSA, BSA, RSA, and fish serum protein [15,16,17,18,19,20,22,23,24,25,26,27,33,34,35].
