# Supplementary material for: Perfluoroalkyl Acid Binding with Peroxisome Proliferator-Activated Receptors α, γ, and δ, and Fatty Acid Binding Proteins by Equilibrium Dialysis with a Comparison of Methods
Source: Toxics. 2021 Feb 26;9(3):45. doi: 10.3390/toxics9030045 (PMC7996760; doi:10.3390/toxics9030045)
Supplement: Supplementary file 1 [file toxics-09-00045-s001.pdf]

toxics

Article

Supplementary Material: Perfluoroalkyl Acid Binding with Peroxisome Proliferator-Activated Receptors α, γ, and δ, and Fatty Acid Binding Proteins by Equilibrium Dialysis with a Comparison of Methods

Manoochehr Khazaee, Emerson Christie, Weixiao Cheng, Mandy Michalsen, Jennifer Field and Carla Ng

Molecular Dynamics Method for Affinity Screening

A previously developed molecular dynamics (MD) workflow [1] was used to estimate protein binding affinities (free energy of binding, ΔG_{bind}) for selected perfluoroalkyl acids (PFAAs). Affinities were subsequently translated to dissociation constants, K_Ds. Briefly, the workflow consists of three major steps: molecular docking, MD simulation, and molecular mechanics combined with Poisson–Boltzmann surface area (MM-PBSA) energy calculation [1]. The MM-PBSA method [2] was used to calculate ΔG_{bind} as follows:

$$\Delta G_{bind} = G^{Complex} - G^{Protein} - G^{PFAS}$$

where $G^{Complex}$, $G^{Protein}$, and G^{PFAS} are the free energies of the protein–PFAA complex, the protein, and the PFAA ligand, respectively. The energy terms were calculated using the *MMPBSA.py* program in AMBER 14. The calculated ΔG_{bind} values were then translated into equilibrium dissociation constants (K_D, with units of μM) using the following equation [3,4]:

$$\Delta G_{bind} = RT\ln (K_D / C_0)$$

where R is the gas constant (1.987 cal K^{-1} mol^{-1}), T is temperature (which is assumed to be 300 K), and C_0 is the standard state concentration (1 M). All simulations were carried out on an AMBER GPU Certified molecular dynamics workstation (Exxact Corporation, Fremont, CA, USA).

Material Extractions for Sorption Quality Control

Dialysis filters and vials (Figure S1) were extracted according to Robel et. al. (2020). Briefly, items were cut into 4.0 ± 0.5 cm^2 pieces with methanol rinsed scissors. Materials were extracted by submerging with 3.3 mL of heated methanol (60–65 °C), shaking on a wrist-action shaker for 10 min, centrifuging at 2808 g for 10 min, and then collecting the supernatant a secondary centrifuge tube. This process was repeated two additional times with each round's supernatant collected in the same secondary tube, yielding a 9.9 mL extract. Extracts were brought to a final volume of 10 mL with additional methanol.

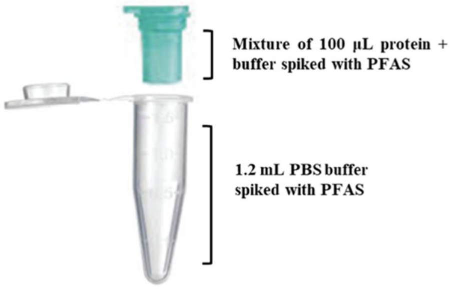

Figure S1. Equilibrium dialysis setup with materials used (dialysis filters and vials) shown.

Material extracts were prepared for analysis as follows: 1) 60 μL aliquots of extract were placed in 1.5 mL HDPE autosampler vials, 2) each vial was spiked with 0.72 ng of isotopically labeled standards, and 3) vials were diluted with methanol to a final volume of 1.2 mL. In order to assess sorption to the dialysis filters and vials, a spike and recovery experiment was performed. Filters and vials were equilibrated on a shaker for 24 h with 1.5 mL of 500 ng/L of native PFASs (Table S1) in water. The spiked water was removed and extracted utilizing the micro liquid-liquid extraction technique described by Backe et. al. [5] and modified by Barzen-Hanson et al. [6].

Molecular Dynamics Results for PFAS-protein Affinity Screening

After the serum albumins, liver fatty acid binding protein (L-FABP) is probably the most-studied protein for binding with per- and polyfluoroalkyl substances (PFAS), both experimentally and using molecular modeling tools [7–10]. The focus on this particular fatty acid binding protein is driven in large part by observations of high accumulation of long-chain PFAS in liver tissue [11]. Existing literature shows a strong increase of binding affinity between PFAS and L-FABP up to a carbon chain length for perfluoroalkyl carboxylic acids (PFCAs) of 11, after which it levels off. In our previous modeling study [1], which established the MD framework used here, perfluorohexanoic acid (PFHxA) was the only short-chain PFCA predicted to bind strongly with L-FABP, but was a clear outlier in the chain length relationship. Here we increased the simulation time in order to sample a greater number of conformations, thus improving our predictions. The updated predictions for all PFCAs now fall in line with the expected chain length trend (Figure 3). The strongest binding was predicted for perfluorooctanoic acid (PFOA), perflorononanoic acid (PFNA), and perfluorooctane sulfonate (PFOS). Among the short-chain PFAS, binding was strongest for perfluorobutanoic aicd (PFBS).

There are no published experimental or modeling studies for PFAS binding with other fatty acid binding proteins, precluding comparisons with our evaluation of intenstinal fatty acid binding protein, I-FABP. Our MD results indicated strongest I-FABP binding affinities for perfluoroheptanoic acid (PFHpA) and PFNA among the carboxylates (Figure 3C), while binding between I-FABP and all sulfonates was predicted to be weak, with no chain length trend and little difference in K_D among them (Figure 3D). This emphasizes the point that PFAS-protein binding affinity is not determined exclusively by PFAS chain length; protein- and PFAS-specific attributes determine binding affinity and should be considered individually.

The relationship between binding affinity predicted by MD and chain length is even weaker for the peroxisome proliferator-activated nuclear receptors, PPARs (Figure 2). In some cases, simulations predict similar or stronger binding for short-chain PFAS than for long-chain PFAS. For example, among the PFCAs, PPAR-α (Figure 2A) is surprisingly predicted to bind most strongly with PFBA. For the remaining PFCAs all binding affinities are relatively weak and overlapping, with K_D values higher than those considered biologically relevant. In comparison, binding with PFSAs is predicted to be relatively stronger, though without a chain length dependence; PPAR-α is predicted to bind equally well with PFBS and PFOS and less strongly with perfluorohexane sulfonate, PFHxS (Figure 2B).

Previous studies found mixed evidence of PPAR-γ activation by PFOA and PFOS. Takacs and Abbott [12] found no evidence of PPAR-γ activation by either PFOA or PFOS (in contrast with PPAR-α), while Vanden Heuvel et al. [13] found that PFOA and PFOS were at least partial activators of PP*AR*-γ, but with lower activity than PPAR alpha. Finally, Buhrke et al. [14] found PFOA activated PPAR-γ in primary human hepatocytes. The predicted binding affinities for PPAR-γ with both PFCAs and PFSAs (Figure 2C and D) were all relatively weak and about the same except for PFNA and PFOS, which were the only ones predicted to have moderate to strong binding (geometric mean $K_D \leq 1$ μM). Finally, the binding affinities predicted for PPAR-δ were strongest for PFPeA among the PFCAs, but all were in the micromolar and larger range (Figure 2E). For PFSAs, binding

was predicted to be only slightly stronger, with essentially no difference in predicted binding affinities among PFSA chain lengths (Figure 2F).

Based on the MD results, a set of 17 PFAS-protein pairs were selected for further investigation by equilibrium dialysis (Table S1).

Table S1. Matrix of Selected Protein–PFAS combinations for batch analysis.

PFAS	L-FABP	I-FABP	PPAR-α	PPAR-γ	PPAR-δ
PFBA			X		X
PFHxA	X		X		
PFHpA		X	X		
PFOA	X			X	
PFNA		X	X		
PFBS	X				X
PFHxS	X				X
PFOS	X			X	X

Additional Dialysis Results

Peroxisome Proliferator-Activated Nuclear Receptors (PPARs)

For PPAR-α, no measurable binding was observed for either PFBA (Figure S2A) despite strong binding predicted by MD. For PFHpA, the lack of observed binding was in agreement with modeling results (Figure S2B).

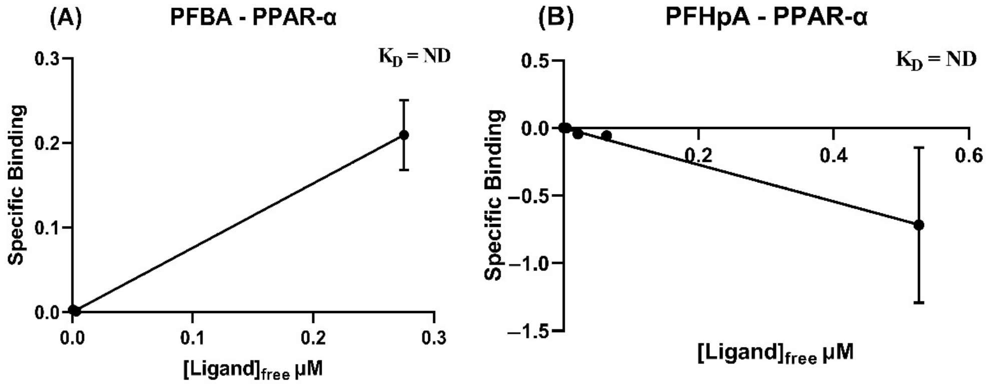

Figure S2. Equilibrium dialysis results for binding affinity of perfluorobutanoic acid, PFBA (**A**) and perfluoroheptanoic acid, PFHpA (**B**) with peroxisome proliferator-activated nuclear receptor, PPAR-α at pH = 7.4 and ionic strength = 18.1 mS/cm. No K_D could be ascertained from these data. The negative result for PFHpA indicates chemical may have been lost from the system due to non-specific interactions that were not due to the protein (e.g. sorption) or that there was a problem with the analysis of PFAS in the dialysate.

For PPAR-γ, binding to PFOA was found to be substantially stronger than for PFOS (Figure S3). PFOS bound more strongly to PPAR-δ (Figure S4A), with a K_D between that of PFOA and PFOS for PPAR-γ. For PFBS, no measurable binding was found for PPAR-δ (Figure S4B).

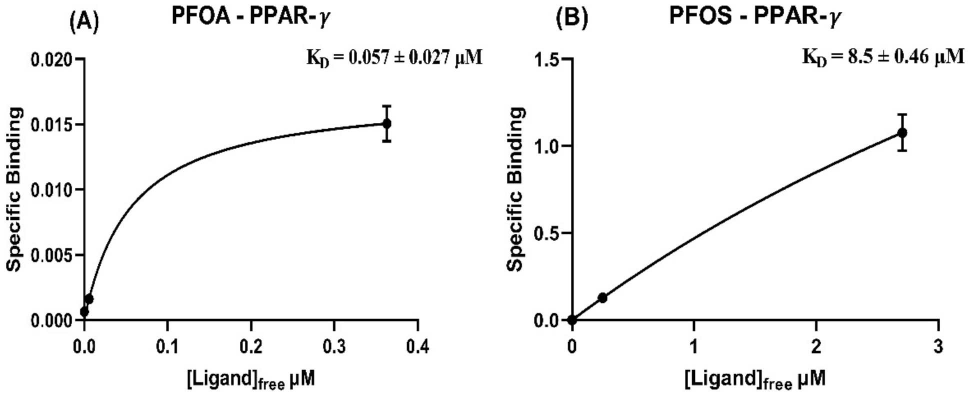

Figure S3. Equilibrium dialysis results for binding affinity of perfluorooctanoic acid, PFOA (**A**) and perfluorooctane sulfonate, PFOS (**B**) with peroxisome proliferator-activated nuclear receptor, PPAR-γ at pH = 7.4 and ionic strength = 18.1 mS/cm.

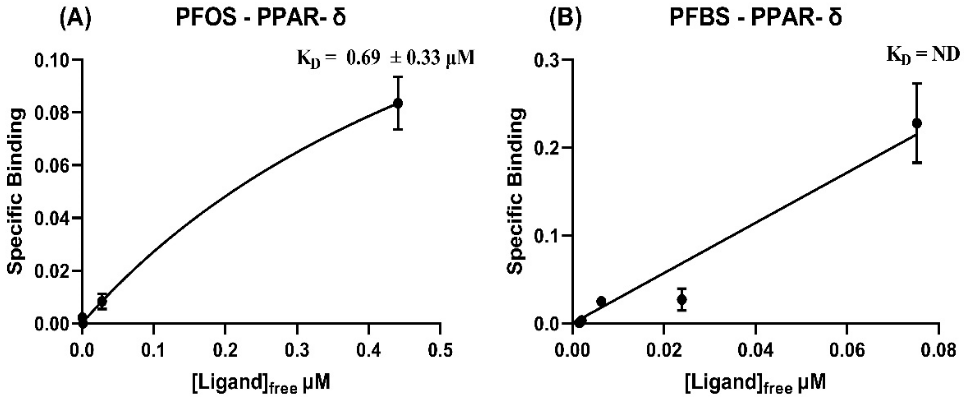

Figure S4. Equilibrium dialysis results for binding affinity of perfluorooctane sulfonate, PFOS (**A**) and perfluorobutane sulfonate, PFBS (**B**) with peroxisome proliferator-activated nuclear receptor, PPAR-δ at pH = 7.4 and ionic strength = 18.1 mS/cm.

Fatty Acid Binding Proteins (FABPs)

For L-FABP, dialysis results agreed with earlier observations of chain-length dependent binding. Long-chain PFAAs (PFOA, PFHxS) bound relatively strongly to L-FABP, whereas short-chain PFAAs (PFBS, PFHxA) had no measurable binding (Figure S5). For I-FABP, the short-chain PFHpA also showed no measurable binding (Figure S5).

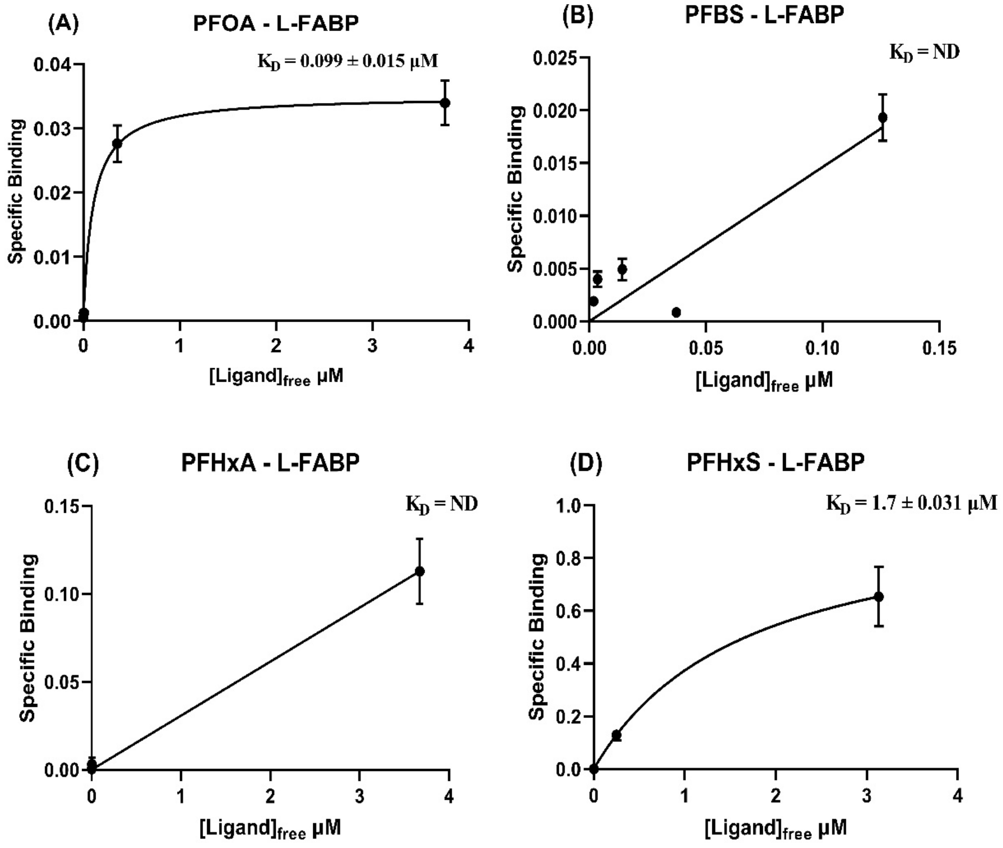

Figure S5. Equilibrium dialysis results for binding affinity of perfluorooctanoic acid, PFOA (**A**), perfluorobutane sulfonate, PFBS (**B**), perfluorohexanoic acid, PFHxA (**C**), and perfluorohexane sulfonate, PFHxS (**D**) with liver fatty acid binding protein, L-FABP, at pH = 7.4 and ionic strength = 18.1 mS/cm.

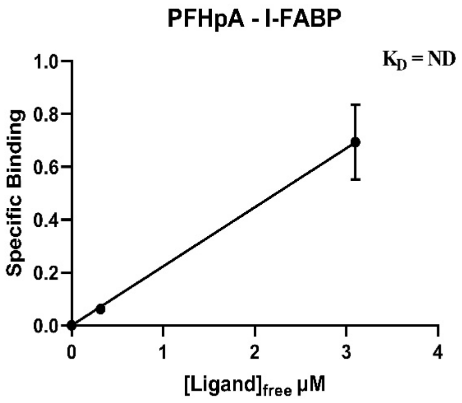

Figure S6. Equilibrium dialysis results for binding affinity of perfluoroheptanoic acid, PFHpA, with intestinal fatty acid binding protein, I-FABP, at pH = 7.4 and ionic strength = 18.1 mS/cm.

Comparison of Published K_Ds for Serum Albumins

A literature review of published studies for measured binding affinities using a wide array of techniques including equilibrium dialysis show values that range over several

orders of magnitude for a single PFAS for both human serum albumin (HSA, Figure S7) and bovine serum albumin (BSA, Figure S8). Data extracted from the literature and used to construct similar comparisons for FABPs and PPAR isoforms, shown in main text Figure 7, are shown in Table S2. Data extracted from the literature and used to construct the comparisons for HSA and BSA are shown in Table S3.

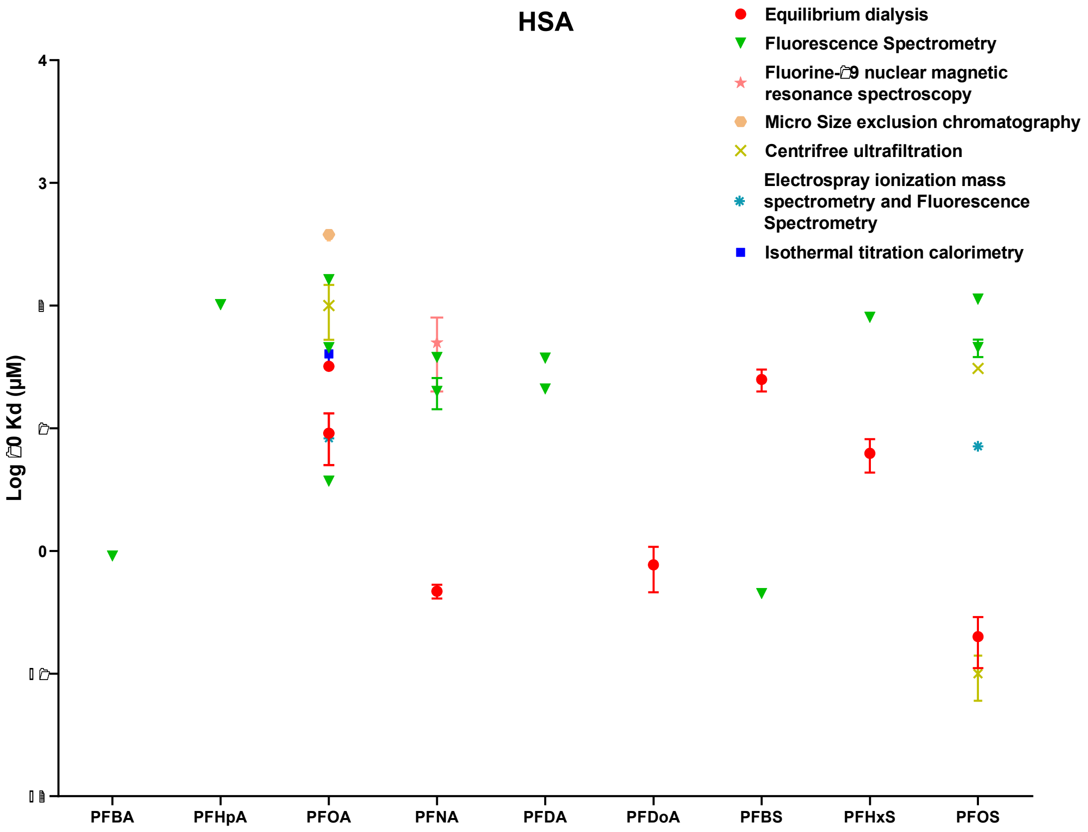

Figure S7. Comparison of reported equilibrium dissociation constant, K_D (± SE) values for human serum albumin, HSA. Data extracted from literature [15–24]. Values plotted as log of K_D, indicating order-of-magnitude differences.

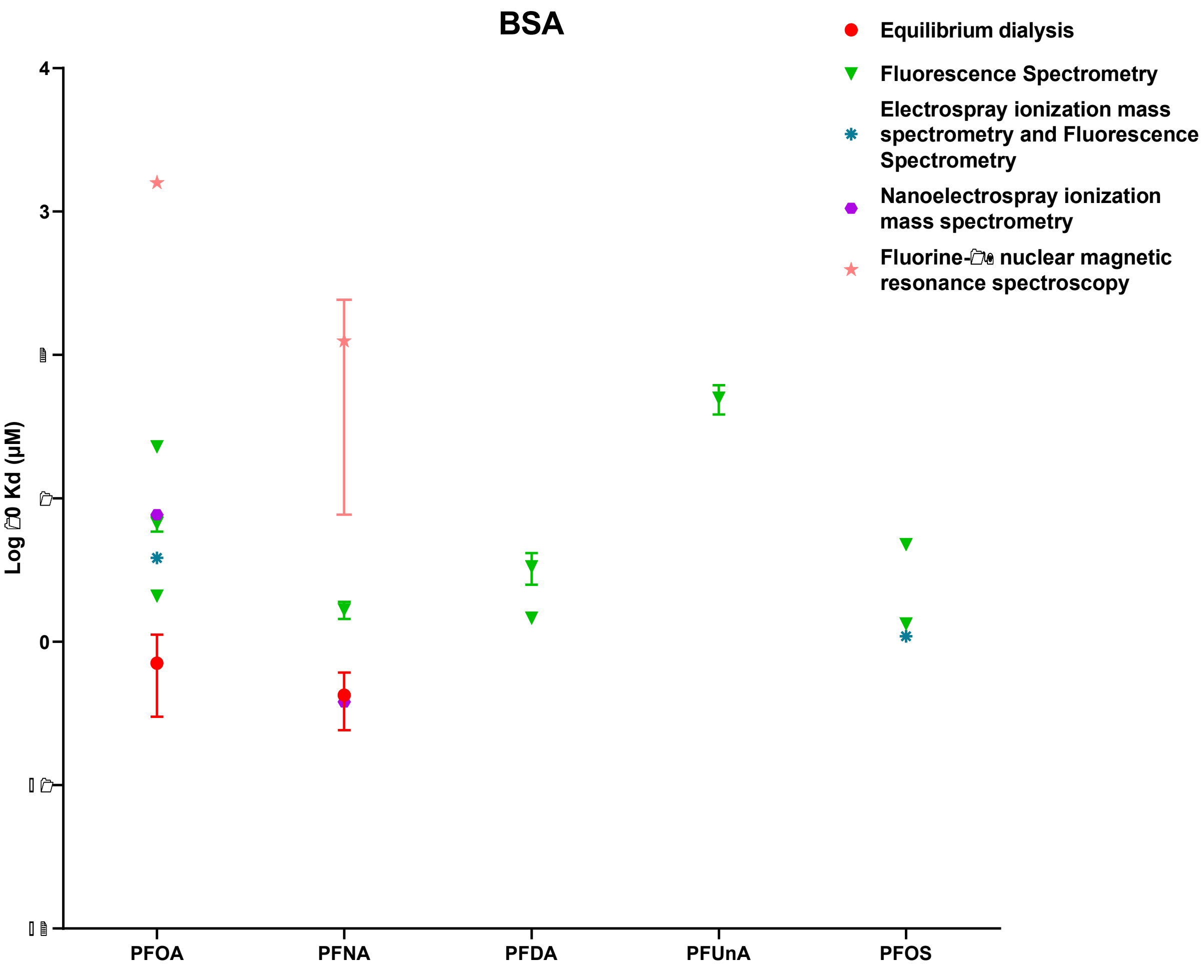

Figure S8. Comparison of reported equilibrium dissociation constant, K_D (± SE) values for bovine serum albumin, BSA. Data extracted from literature [15,20,24–27]. Values plotted as log of K_D, indicating order-of-magnitude differences.

Table S2. Comparison of K_D from different methods for L- and I-FABP and PPAR-α, γ, and δ

Protein	PFAS	Kd (μM)						IC50 (μM)			
		Equilibrium Dialysis		Isothermal Titration Calorimetry (ITC)		Fluorescence Displacement		Competitive Binding Assay		Fluorescence Displacement	
		Kd	Reference	Kd	Reference	Kd	Reference	Kd	Reference	Kd	Reference
L-FABP	PFHxA	ND*	Current study			ND	[8]			ND	
						261.7 (rat)	[28]				
	PFOA	0.099	Current study	6.49	[9]	50.4	[8]			9	[8]
						2.36	[9]			8.14	[9]
						8.03	[32]			2.15	[32]
						13.14 (rat)	[28]				
	PFBS	ND	Current study			1034	[8]			185	[8]
	PFHxS	1.695	Current study			85.7	[8]			15.3	[8]
	PFOS	0.184	Current study			18.5	[8]			3.3	[8]
						4.99	[32]			1.34	[32]
	PFBA					ND	[8]			ND	[8]

	PFPeA					336	[8]			ND	[8]
	PFNA			3.14	[9]	1.32	[9]			4.55	[9]
						16.2	[8]			2.9	[8]
	PFDA					12.9	[8]			2.3	[8]
	PFUnDA					10.6	[8]			1.9	[8]
	PFDoDA					12.3	[8]			2.2	[8]
	PFTeDA					60.5	[8]			10.8	[8]
	PFHxDA					115.4	[8]			20.6	[8]
	PFOcDA					62.2	[8]			11.1	[8]
	6:2 FTOH					ND	[8]			ND	[8]
	8:2 FTOH					ND	[8]			ND	[8]
	6:2 FTCA					436.55	[32]			116.88	[32]
	6:2 FTSA					345.54	[32]			78.97	[32]
	6:2 Cl-PFESA					4.05	[32]			1.14	[32]
I-FABP	PFHpA	ND	Current study								
	PFNA	ND	Current study								
PPAR-α	PFBA	ND	Current study					3224	[29]		
	PFHxA	0.097	Current study					904	[29]		
	PFHpA	ND	Current study					275	[29]		
	PFPeA							3279	[29]		
	PFOA							371	[29]		
	PFDA							366	[29]		
	PFUnDA							3265	[29]		
	PFBS							7745	[29]		
	PFHxS							140	[29]		
	PFOS							237	[29]		
	PFNA	0.083	Current study					277	[29]		
PPAR-γ	PFOA	0.057	Current study			300.9	[30]			43.5	[30]
	PFOS	8.47	Current study			93.7	[30]			13.5	[30]
	PFBA					ND	[30]			ND	[30]
	PFHxA					ND	[30]			ND	[30]
	PFHpA					1330.4	[30]			192.4	[30]
	PFNA					155.4	[30]			22.4	[30]
	PFDA					8.4	[30]			12.2	[30]
	PFUnDA					58.2	[30]			8.4	[30]
	PFDoDA					143.1	[30]			20.6	[30]
	PFTeDA					157.8	[30]			22.8	[30]
	PFHxDA					128.2	[30]			18.5	[30]
	PFOcDA					107.6	[30]			15.5	[30]
	PFBS					ND	[30]			ND	[30]
	PFHxS					285.3	[30]			41.2	[30]
	6:2 FTOH					ND	[30]			ND	[30]
	8:2 FTOH					ND	[30]			ND	[30]
PPAR-δ	PFBA	0.044	Current study					ND	[31]		
	PFBS	ND	Current study					ND	[31]		
	PFHxS	0.035	Current study					ND	[31]		
	PFOS	0.686	Current study					76.9	[31]		
	PFHxA							ND	[31]		
	PFHpA							ND	[31]		
	PFOA							ND	[31]		
	PFNA							127.9	[31]		
	PFDA							56.6	[31]		

PFUnDA	47.7	[31]
PFDoA	32.6	[31]
PFTrDA	52.6	[31]
PFTeDA	110.8	[31]
PFHxDA	159.8	[31]
PFOcDA	ND	[31]
6:2 FTOH	ND	[31]
8:2 FTOH	ND	[31]

*ND indicates non-detect (no measurable binding).

Table S3. Comparison of K_D from different methods for HSA, BSA, RSA, and fish serum proteins.

Protein	PFAS	Kd (μM)															
		**Equilibrium Dialysis**		**Nanoelectrospray Ionization Mass Spectrometry**		**Fluorescence Spectrometry**		**Fluorine-19 Nuclear Magnetic Resonance Spectroscopy**		**Micro Size Exclusion Chromatography**		**Centrifree Ultrafiltration**		**Electrospray Ionization Mass Spectrometry and Fluorescence Spectrometry**		**Isothermal Titration Calorimetry (ITC)**	
		**Kd**	**Reference***	**Kd**	**Reference***	**Kd**	**Reference**	**Kd**	**Reference***	**Kd**	**Reference***	**Kd**	**Reference***	**Kd**	**Reference***	**Kd**	**Reference***
HSA	PFNA	0.47	[15]			20	[20]	50	[20]								
						37.88	[19]										
	PFOA	32.05	[16]			3.7	[17]			380	[21]	100	[22]	8.3	[24]	40.48	[16]
		9.1	[34]			45.24	[19]										
		9.1	[33]			162.6 (296 K)	[18]										
	PFDA					20.94	[19]										
						37.33 (296 K)	[18]										
	PFBA	6.66	[33]			0.91 (site I)	[17]										
						ND (slight effect)	[18]										
	PFHxA					ND (slight effect)	[18]										
	PFOS	2	[33]			113	[19]					30.7	[23]	7.14	[24]		
						45.45 (trp site) –0.13 (site II)	[17]					0.08	[22]				
	PFDoDA	0.77	[33]			101.73	[19]										
						0.83 (site II)	[17]										
	PFBS					0.45 (site I)–0.15 (site II)	[17]										
	PFHxS	6.25	[33]			80.13	[19]										
	PFHpA					101.73	[19]										
	PFBS	25	[33]														
	6:2 Cl-PFESA											16.7	[23]				
BSA	PFOA	0.71	[15]	7.69	[15]	2.08 (295 K)	[25]	1587.3	[20]					3.84	[24]		
						0.6	[20]										
						22.93 (300 K)	[26]										
	PFNA	0.425†	[15]	0.38	[15]	1.67	[20]	125	[20]								

	PFDA	3.33	[20]						
		1.46 (300 K)	[26]						
	PFOS	4.76 (294 K)	[27]					1.09	[24]
		1.33 (295 K)	[25]						
	PFUnDA	50	[20]						
RSA	PFOA			290	[21]	360	[21]		
Fish serum protein	PFHxS	869	[35]						
	PFOS	643	[35]						
	PFOA	6370	[35]						
	PFNA	2590	[35]						
	PFDA	1858	[35]						
	PFUnDA	1300	[35]						
	PFDoDA	1210	[35]						

* See main SI document for full references. † Average of two values.

References

1. Cheng, W.; Ng, C.A. Predicting Relative Protein Affinity of Novel Per- and Polyfluoroalkyl Substances (PFASs) by An Efficient Molecular Dynamics Approach. *Environ. Sci. Technol.* **2018**, *52*, 7972–7980, doi:10.1021/acs.est.8b01268.
2. Miller, B.R.; McGee, T.D.; Swails, J.M.; Homeyer, N.; Gohlke, H.; Roitberg, A.E. MMPBSA.Py: An Efficient Program for End-State Free Energy Calculations. *J Chem Theory Comput* **2012**, *8*, 3314–3321, doi:10.1021/ct300418h.
3. Kastritis, P.L.; Bonvin, A.M.J.J. On the Binding Affinity of Macromolecular Interactions: Daring to Ask Why Proteins Interact. *J R Soc Interface* **2013**, *10*, 20120835, doi:10.1098/rsif.2012.0835.
4. Caldwell, G.W.; Yan, Z. Isothermal Titration Calorimetry Characterization of Drug-Binding Energetics to Blood Proteins. In *Optimization in Drug Discovery:* In Vitro *Methods*; Yan, Z., Caldwell, G.W., Eds.; Methods in Pharmacology and Toxicology; Humana Press: Totowa, NJ, USA, 2004; pp. 123–149 ISBN 978-1-59259-800-7.
5. Backe, W.J.; Day, T.C.; Field, J.A. Zwitterionic, Cationic, and Anionic Fluorinated Chemicals in Aqueous Film Forming Foam Formulations and Groundwater from U.S. Military Bases by Nonaqueous Large-Volume Injection HPLC-MS/MS. *Environ. Sci. Technol.* **2013**, *47*, 5226–5234, doi:10.1021/es3034999.
6. Barzen-Hanson, K.A.; Roberts, S.C.; Choyke, S.; Oetjen, K.; McAlees, A.; Riddell, N.; McCrindle, R.; Ferguson, P.L.; Higgins, C.P.; Field, J.A. Discovery of 40 Classes of Per- and Polyfluoroalkyl Substances in Historical Aqueous Film-Forming Foams (AFFFs) and AFFF-Impacted Groundwater. *Environ. Sci. Technol.* **2017**, *51*, 2047–2057, doi:10.1021/acs.est.6b05843.
7. Luebker, D.J.; Hansen, K.J.; Bass, N.M.; Butenhoff, J.L.; Seacat, A.M. Interactions of Flurochemicals with Rat Liver Fatty Acid-Binding Protein. *Toxicology* **2002**, *176*, 175–185, doi:10.1016/S0300-483X(02)00081-1.
8. Zhang, L.; Ren, X.-M.; Guo, L.-H. Structure-Based Investigation on the Interaction of Perfluorinated Compounds with Human Liver Fatty Acid Binding Protein. *Environ. Sci. Technol.* **2013**, *47*, 11293–11301, doi:10.1021/es4026722.
9. Sheng, N.; Li, J.; Liu, H.; Zhang, A.; Dai, J. Interaction of Perfluoroalkyl Acids with Human Liver Fatty Acid-Binding Protein. *Arch Toxicol* **2016**, *90*, 217–227, doi:10.1007/s00204-014-1391-7.
10. Yang, D.; Han, J.; Hall, D.R.; Sun, J.; Fu, J.; Kutarna, S.; Houck, K.A.; LaLone, C.A.; Doering, J.A.; Ng, C.A.; et al. Nontarget Screening of Per- and Polyfluoroalkyl Substances Binding to Human Liver Fatty Acid Binding Protein. *Environ. Sci. Technol.* **2020**, *54*, 5676–5686, doi:10.1021/acs.est.0c00049.
11. Ng, C.A.; Hungerbühler, K. Bioaccumulation of Perfluorinated Alkyl Acids: Observations and Models. *Environ. Sci. Technol.* **2014**, *48*, 4637–4648, doi:10.1021/es404008g.
12. Takacs, M.L.; Abbott, B.D. Activation of Mouse and Human Peroxisome Proliferator–Activated Receptors (α, β/δ, γ) by Perfluorooctanoic Acid and Perfluorooctane Sulfonate. *Toxicol Sci* **2007**, *95*, 108–117, doi:10.1093/toxsci/kfl135.
13. Heuvel, J.V.; Thompson, J.; Frame, S.; Gillies, P. Differential Activation of Nuclear Receptors by Perfluorinated Fatty Acid Analogs and Natural Fatty Acids: A Comparison of Human, Mouse, and Rat Peroxisome Proliferator-Activated Receptor-α, -β, and -γ, Liver X Receptor-β, and Retinoid X Receptor-α. *Toxicol. Sci.* **2006**, *92*, 476–489.
14. Buhrke, T.; Krüger, E.; Pevny, S.; Rößler, M.; Bitter, K.; Lampen, A. Perfluorooctanoic Acid (PFOA) Affects Distinct Molecular Signalling Pathways in Human Primary Hepatocytes. *Toxicology* **2015**, *333*, 53–62, doi:10.1016/j.tox.2015.04.004.
15. Bischel, H.N.; MacManus-Spencer, L.A.; Luthy, R.G. Noncovalent Interactions of Long-Chain Perfluoroalkyl Acids with Serum Albumin. *Environ. Sci. Technol.* **2010**, *44*, 5263–5269, doi:10.1021/es101334s.
16. Wu, L.-L.; Gao, H.-W.; Gao, N.-Y.; Chen, F.-F.; Chen, L. Interaction of Perfluorooctanoic Acid with Human Serum Albumin. *BMC Structural Biology* **2009**, *9*, 31, doi:10.1186/1472-6807-9-31.
17. Chen, Y.-M.; Guo, L.-H. Fluorescence Study on Site-Specific Binding of Perfluoroalkyl Acids to Human Serum Albumin. *Arch Toxicol* **2009**, *83*, 255–261, doi:10.1007/s00204-008-0359-x.
18. Chen, H.; Wang, Q.; Cai, Y.; Yuan, R.; Wang, F.; Zhou, B. Investigation of the Interaction Mechanism of Perfluoroalkyl Carboxylic Acids with Human Serum Albumin by Spectroscopic Methods. *Int. J. Environ. Res. Public Health* **2020**, *17*, 1319, doi:10.3390/ijerph17041319.
19. Hebert, P.C.; MacManus-Spencer, L.A. Development of a Fluorescence Model for the Binding of Medium- to Long-Chain Perfluoroalkyl Acids to Human Serum Albumin Through a Mechanistic Evaluation of Spectroscopic Evidence. *Anal. Chem.* **2010**, *82*, 6463–6471, doi:10.1021/ac100721e.
20. MacManus-Spencer, L.A.; Tse, M.L.; Hebert, P.C.; Bischel, H.N.; Luthy, R.G. Binding of Perfluorocarboxylates to Serum Albumin: A Comparison of Analytical Methods. *Anal. Chem.* **2010**, *82*, 974–981, doi:10.1021/ac902238u.
21. Han, X.; Snow, T.A.; Kemper, R.A.; Jepson, G.W. Binding of Perfluorooctanoic Acid to Rat and Human Plasma Proteins. *Chem. Res. Toxicol.* **2003**, *16*, 775–781, doi:10.1021/tx034005w.
22. Beesoon, S.; Martin, J.W. Isomer-Specific Binding Affinity of Perfluorooctanesulfonate (PFOS) and Perfluorooctanoate (PFOA) to Serum Proteins. *Environ. Sci. Technol.* **2015**, *49*, 5722–5731, doi:10.1021/es505399w.
23. Sheng, N.; Wang, J.; Guo, Y.; Wang, J.; Dai, J. Interactions of Perfluorooctanesulfonate and 6:2 Chlorinated Polyfluorinated Ether Sulfonate with Human Serum Albumin: A Comparative Study. *Chem. Res. Toxicol.* **2020**, *33*, 1478–1486, doi:10.1021/acs.chemrestox.0c00075.
24. Chi, Q.; Li, Z.; Huang, J.; Ma, J.; Wang, X. Interactions of Perfluorooctanoic Acid and Perfluorooctanesulfonic Acid with Serum Albumins by Native Mass Spectrometry, Fluorescence and Molecular Docking. *Chemosphere* **2018**, *198*, 442–449, doi:10.1016/j.chemosphere.2018.01.152.

25. Chen, H.; He, P.; Rao, H.; Wang, F.; Liu, H.; Yao, J. Systematic Investigation of the Toxic Mechanism of PFOA and PFOS on Bovine Serum Albumin by Spectroscopic and Molecular Modeling. *Chemosphere* **2015**, *129*, 217–224, doi:10.1016/j.chemosphere.2014.11.040.
26. Qin, P.; Liu, R.; Pan, X.; Fang, X.; Mou, Y. Impact of Carbon Chain Length on Binding of Perfluoroalkyl Acids to Bovine Serum Albumin Determined by Spectroscopic Methods. *J. Agric. Food Chem.* **2010**, *58*, 5561–5567, doi:10.1021/jf100412q.
27. Li, L.; Song, G.W.; Xu, Z.S. Study on the Interaction Between Bovine Serum Albumin and Potassium Perfluoro Octane Sulfonate. *J. Dispers. Sci. Technol.* **2010**, *31*, 1547–1551, doi:10.1080/01932690903294139.
28. Woodcroft, M.W.; Ellis, D.A.; Rafferty, S.P.; Burns, D.C.; March, R.E.; Stock, N.L.; Trumpour, K.S.; Yee, J.; Munro, K. Experimental Characterization of the Mechanism of Perfluorocarboxylic Acids' Liver Protein Bioaccumulation: The Key Role of the Neutral Species. *Environ. Toxicol. Chem.* **2010**, *29*, 1669–1677, doi:10.1002/etc.199.
29. Ishibashi, H.; Hirano, M.; Kim, E.-Y.; Iwata, H. In Vitro and In Silico Evaluations of Binding Affinities of Perfluoroalkyl Substances to Baikal Seal and Human Peroxisome Proliferator-Activated Receptor α. *Environ. Sci. Technol.* **2019**, *53*, 2181–2188, doi:10.1021/acs.est.8b07273.
30. Zhang, L.; Ren, X.-M.; Wan, B.; Guo, L.-H. Structure-Dependent Binding and Activation of Perfluorinated Compounds on Human Peroxisome Proliferator-Activated Receptor γ. *Toxicol. Appl. Pharmacol.* **2014**, *279*, 275–283, doi:10.1016/j.taap.2014.06.020.
31. Li, C.-H.; Ren, X.-M.; Cao, L.-Y.; Qin, W.-P.; Guo, L.-H. Investigation of Binding and Activity of Perfluoroalkyl Substances to the Human Peroxisome Proliferator-Activated Receptor β/δ. *Environ. Sci.: Processes Impacts* **2019**, *21*, 1908–1914, doi:10.1039/C9EM00218A.
32. Sheng, N.; Cui, R.; Wang, J.; Guo, Y.; Wang, J.; Dai, J. Cytotoxicity of Novel Fluorinated Alternatives to Long-Chain Perfluoroalkyl Substances to Human Liver Cell Line and Their Binding Capacity to Human Liver Fatty Acid Binding Protein. *Arch Toxicol* **2018**, *92*, 359–369, doi:10.1007/s00204-017-2055-1.
33. Ulrich, J. A Systematic Investigation of the Effects of Chain Length and Ionic Head Group on Perfluoroalkyl Acid Binding to Human Serum Albumin. *Undergraduate Honors Thesis, Union College, Schenectady, NY.* **2017**, pp 258. Available from: https://digitalworks.union.edu/cgi/viewcontent.cgi?article=1258&context=theses, accessed on 22 February 2021.
34. Morris, M. Investigation of the Mechanism of Binding of Perfluoroalkyl Acids with Human Serum Albumin Using an Improved Approach to Equilibrium Dialysis. *Undergraduate Honors Thesis, Union College, Schenectady, NY.* **2014**, pp 68. Available from: https://digitalworks.union.edu/cgi/viewcontent.cgi?article=1561&context=theses, accessed on 22 February 2021.
35. Zhong, W.; Zhang, L.; Cui, Y.; Chen, M.; Zhu, L. Probing Mechanisms for Bioaccumulation of Perfluoroalkyl Acids in Carp (Cyprinus Carpio): Impacts of Protein Binding Affinities and Elimination Pathways. *Sci. Total Environ.* **2019**, *647*, 992–999, doi:10.1016/j.scitotenv.2018.08.099.
